# Microbial co-occurrence patterns and community assembly in seamount sediment cores: disentangling the effects of assembly processes on β-diversity

**DOI:** 10.1128/aem.00732-26

**Published:** 2026-06-18

**Authors:** Tao Li, Weidong Luo, Ziya Lin, Wei Xie, Jiao Zhou

**Affiliations:** 1 Guangzhou Marine Geological Survey, China Geological Survey 74717, Guangzhou, China; 2 Key Laboratory of Marine Mineral Resources, Ministry of Natural Resources, Guangzhou Marine Geological Survey 74717, Guangzhou, China; 3 School of Marine Sciences/Research Center of Ocean Climate, Sun Yat-sen University & Southern Marine Science and Engineering Guangdong Laboratory (Zhuhai) 212170, Zhuhai, China; University of Delaware, Lewes, Delaware, USA

**Keywords:** co-occurrence network, community assembly, β-diversity, seamount, sediment core

## Abstract

Seamounts are hotspots of seafloor biodiversity in the open sea, and microorganisms are the base of seamount food webs. However, the mechanism shaping the vertical distribution patterns of microbial community composition in seamount sediment cores remains elusive. Here, we analyzed the microbial communities in seven sediment cores collected from a seamount in the Western Philippine Ocean and its adjacent valley. We investigated the assembly of bacterial and archaeal communities in three sediment cores that contained complete profiles of microbial communities and deciphered how changes in community assembly processes altered β-diversity. In addition, we explored the microbial co-occurrence patterns in the three cores and their correlation with environmental variables. The results revealed that bacterial community assembly was dominated by drift, dispersal limitation, homogenizing dispersal, and homogeneous selection, whereas archaeal community assembly was dominated by drift. Besides, the variability of microbial co-occurrence patterns among the three sediment cores was driven mainly by trace elements. This study supported that dispersal limitation led to a higher β-diversity, whereas homogeneous selection, homogenizing dispersal, and drift resulted in a relatively lower β-diversity. Overall, this study offers key insights into the microbial occurrence patterns and community assembly in seamount sediment cores, and illustrates how different community assembly processes affect β-diversity.

**IMPORTANCE:**

β-Diversity (site-to-site variation in species composition) is important for understanding the mechanisms that generate and maintain biodiversity, but the origin of β-diversity is still largely unknown. Variations in β-diversity can be related to changes in assembly processes, but there is still a lack of a quantitative evaluation for the effects of different assembly processes on β-diversity. Here, we investigated the microbial community assembly in three sediment cores collected from a seamount in the Western Philippine Ocean and its adjacent valley. We compared the differences in β-diversity among bacterial communities that were driven by different assembly processes and found that β-diversity was enhanced by dispersal limitation but decreased by homogeneous selection, homogenizing dispersal, and drift. Our findings underscore the differential impacts of assembly processes on β-diversity, which advances the understanding of the origin of β-diversity.

## INTRODUCTION

Seamounts, defined as undersea mountains that rise steeply from the ocean bottom to below sea level (1), are widely distributed throughout the world’s oceans. It is estimated that there are more than 100,000 seamounts with elevation exceeding 1,000 m (2), and more than 60% of the large seamounts are located in the Pacific Ocean (3). Seamounts are unique compared with other habitats for the following two reasons: (i) they may function as “island groups” or “chains,” resulting in high levels of endemism (4, 5), and (ii) they are biodiversity hot spots (6–8), where species richness is usually several times greater than that in the surrounding seafloor. Microbes play a critical role in the functioning of the seamount ecosystem. Many studies have reported microbial diversity and spatial distribution in surface sediments from seamount areas (9–15); however, the understanding of seamount microbial ecology is limited by the lack of studies describing the vertical distribution patterns and driving forces of microbial communities in sediment cores.

Ecologists have developed methodologies to disentangle the relative importance of deterministic and stochastic processes in shaping natural communities. Stegen’s framework (16) identifies five assembly processes: homogeneous selection (HoS), heterogeneous selection (HeS), dispersal limitation (DL), homogenizing dispersal (HD), and drift (DF) (and speciation). HoS and HeS are two different forms of selection, representing the process of selection under homogeneous and heterogeneous environmental conditions, respectively (17, 18). Dispersal is classified into two categories: DL and HD. The former indicates the restricted movement to a new location and colonization by individuals, whereas the latter denotes a very high rate of dispersal among locations (17–19). Drift refers to random changes in the relative abundances of different species due to stochastic processes such as growth and death (18, 19). Many studies have demonstrated that the spatial patterns of microbial communities are determined mainly by the relative importance of deterministic and stochastic processes (20, 21). Assembly processes may also affect the vertical distribution patterns of microbial communities; however, it has received little attention. Recently, Xiao et al. (22) reported microbial community assembly in sediment cores collected from marine trenches, unraveling the assembly mechanisms in subsurface sedimentary environments.

Co-occurrence networks are widely used to explore species interactions (23), although ecological inferences are criticized by some researchers, who argue that co-occurrence relationships cannot be directly interpreted as ecological interactions (24, 25). Networks comprise nodes and edges, with nodes representing individual taxa and edges representing pairwise associations between taxa. One of the most important benefits of co-occurrence networks is the prediction of the stability of ecological networks (26). For example, keystone taxa, which are identified from node centralities or node connectedness (27, 28), may drive community composition and function (29). Modularity, defined as the number of edges falling within groups minus the expected number in an equivalent random network (30), may have stabilizing effects on the ecological network (31). However, co-occurrence relationships are affected not only by species interactions but also by environmental preferences (32, 33). Lima-Mendez et al. (34) reported that more than one-third of global plankton associations are driven by environmental conditions instead of species interactions. Other researchers have emphasized the correlation between environmental factors (e.g., geographic, climatic, and physicochemical variables) and network topological properties (35–37), providing insights into understanding co-occurrence patterns driven by the environment. An open question is the quantitative assessment of the relative importance of species interactions and environmental factors to co-occurrence patterns.

β-Diversity, defined as site-to-site variation in species composition in a geographical area (38, 39), is crucial for understanding the mechanisms that generate and maintain biodiversity (40–42). The following three hypotheses have been proposed explaining the origin of β-diversity: (i) species composition is uniform across space, and species interactions are crucial; (ii) species composition changes randomly, and differences in species composition are created through limited dispersal of species from the species pool, likely coupled with local speciation; and (iii) patterns of species composition are controlled by environmental conditions (43, 44). Some studies have demonstrated that HeS (45, 46) or environmental heterogeneity (47) promotes the increase in β-diversity, supporting the third hypothesis. Other studies have attributed the increase in β-diversity to the contribution of DL (48–50), supporting the second hypothesis. Zhang et al. (51) associated regional-scale variations in β-diversity with local assembly processes and concluded that HeS and DL cause a higher β-diversity, while HoS and HD lead to a lower β-diversity. β-Diversity may also be affected by the size of the regional species pool (γ-diversity) at regional and larger scales (48, 52, 53), whereas it is not necessary to consider the effect of γ-diversity at local scales.

In this study, we hypothesized that β-diversity is enhanced by HeS and DL but decreased by HD, DF, and HoS at local scales. To address this hypothesis, we investigated the assembly of bacterial and archaeal communities in three sediment cores collected from a seamount in the Western Philippine Ocean (WPB) and its adjacent valley. We compared the differences in β-diversity among local communities driven by different assembly processes. If an assembly process is significantly associated with a higher β-diversity, it may enhance the β-diversity, whereas if a process is significantly associated with a relatively lower β-diversity, it likely decreases the β-diversity. Through this method, we provided direct evidence for how variations in assembly processes alter β-diversity. In addition, we analyzed the variability of microbial co-occurrence patterns among the three sediment cores and the main driving forces.

## MATERIALS AND METHODS

### Study area and sampling

**Fig 1:**
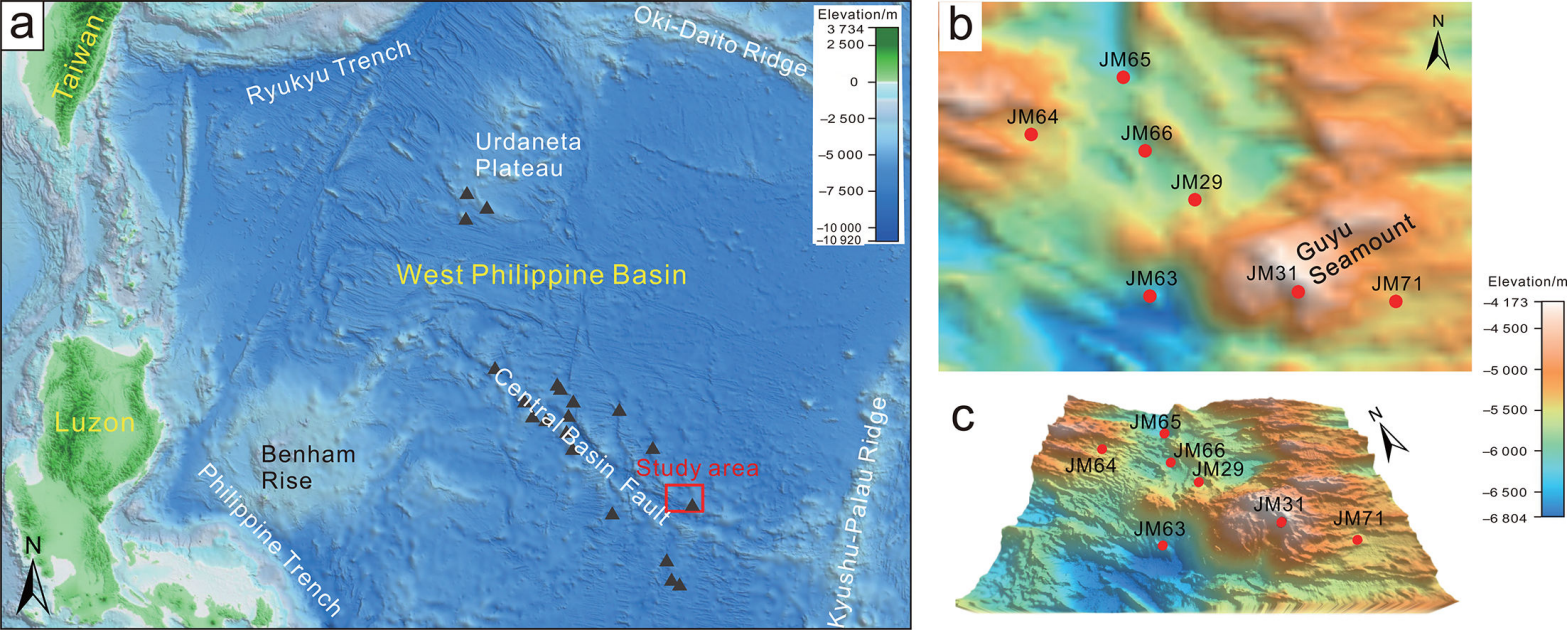
Maps showing the location of the study area in the West Philippine Basin (**a**), the distribution of sampling cores (**b**), and the three-dimensional (3D) morphology of the study area (**c**). The geographic map (**a**) is drawn with the GEBCO_2025 Grid data set (56); triangles denote seamounts. For a more comprehensive description of these seamounts, we refer the reader to a previous study (55).

The WPB is located in the western part of the Philippine Sea, with the average depth varying from 5,500 to 6,000 m (54). The WPB is bounded by the Kyushu-Palau Ridge and the Oki-Daito Ridge to the east, by the Ryukyu Trench to the north, and by the Philippine Trench to the west (see Fig. 1a). Two plateaus, the Benham Rise and the Urdaneta Plateau, are situated on the western part of the WPB. A NW–SE elongated fault, the Central Basin Fault (CBF), spreads in the central area, and many seamounts are formed along the CBF (55) (Fig. 1a). A seamount named Guyu is situated on the southwest side of the CBF. Fieldwork was conducted during two cruises: one in 2019 and another in 2020. Cores JM29, JM71, and JM31 were collected from the bottom, hillside, and summit areas of the Guyu Seamount, respectively; core JM64 was collected from the bottom of an unknown submarine mountain; and cores JM63, JM65, and JM66 were collected from the middle valley (Table S1; Fig. 1b and c). All the cores were sealed and frozen onboard at −80°C. After they were transported to the laboratory, the uppermost 20 cm of each core was sliced into 2-cm-thick intervals. One half (~5 g) of each sample was used for DNA extraction, and the other half (~5 g) was used for physicochemical analyses. However, approximately half of the samples were discarded because of DNA extraction failure.

### Measurement of physicochemical parameters

Grain size, major elements, minor elements, organic carbon (OC), and organic nitrogen (ON) were measured in the sediment samples. The sediments were pretreated with 10% HCl and 10% H_2_O_2_ to remove carbonates and organic carbon, after which a Mastersizer 2000 laser particle size analyzer (Malvern Instruments Ltd., U.K.) was used to measure the grain size. Major elements (e.g., Na_2_O, Al_2_O_3_, Fe_2_O_3_, CaO, MgO, K_2_O, MnO, and P_2_O_5_) were detected using an Axios X-ray fluorescence spectrometer (PANalytical, Eindhoven, the Netherlands). An inductively coupled plasma mass spectrometry (ICP‒MS, Agilent 7900, Agilent Technologies Japan, Ltd., Japan) was used to measure the concentrations of trace elements such as Cd, Co, Cr, Cu, Mo, Ni, Pb, V, and Zn, while As was detected using an atomic fluorescence spectrophotometer (AFS-930, Beijing Jitian Instruments, Beijing, China). The contents of OC and ON were determined via an organic elemental analyzer (Elementar Vario Macro cube, Germany). However, physicochemical measurements were not available for the upper layers (0–6 cm) of cores JM63, JM65, and JM71 because the entire samples were used for DNA extractions. One-way analysis of variance (ANOVA) was used to test differences in physicochemical variables between the seamount and submarine valley.

### Environmental DNA extraction, PCR amplification, and high-throughput sequencing

Sediment samples (5~10 g) were used to extract DNA (Table S1) with a FastDNA Spin Kit (MP Biomedicals, USA) according to the manufacturer’s manual. The V4 region of the 16S rRNA gene (~250 nucleotides) was amplified using the general prokaryotic primers 515F (5′-GTGCCAGCMGCCGCGGTAA-3′) and 806R (5′-GGACTACHVGGGTWTCTAAT-3′) (57) with barcodes. This primer pair is among the best candidates for identifying both bacteria and archaea (58). PCR was performed in 50 µL reactions consisting of 3 μL of DNA template (~5 ng/µL), 25 μL of 2× Premix Taq (Takara Biotechnology, Dalian Co. Ltd., China), and 1 μL of each primer (10 mM). Thermal cycling was performed on a BioRad S1000 thermal cycler (Bio-Rad Laboratory) with the following program: initial denaturation at 94°C for 5 min; 30 cycles of denaturation at 94°C for 30 s, annealing at 52°C for 30 s, and elongation at 72°C for 30 s; and a final elongation at 72°C for 10 min. The resulting PCR product was subsequently sequenced on an Illumina MiSeq PE300 platform (commissioned by Guangdong Magigene Biotechnology).

Following demultiplexing, paired-end sequences were merged via FLASH v1.2.11 (version 1.2.11) (59). Low-quality reads (average quality score <20) were filtered out using fastp (version 0.20.0) (60), and the remaining high-quality reads were denoised using the DADA2 (61) plugin in the QIIME 2 pipeline (version 2020.2) (62). The sequences were clustered into operational taxonomic units (OTUs) with a 97% similarity cutoff (broadly equivalent to the species level) using UPARSE and taxonomically assigned on the basis of the SILVA database (version 138.2). The sequences were rarefied to 78,920 per sample.

### Network analyses

The microbial OTU data were normalized by the transfer matrix method, and a co-occurrence network was generated on the basis of the Pearson correlation matrix using the R package *ggClusterNet* (version 0.1.0) (63). Only OTUs that occurred in at least 10 communities were incorporated into the network analysis, according to Shi et al. (64). The correlation cutoff was determined to be 0.6 (65, 66), and a *P*-value <0.05 was used as the cutoff for statistical significance. The network was divided into modules using the fast greedy algorithm in the R package *igraph* (version 2.0.3), and both the network and modules were visualized using Gephi software (version 0.10.1). On the basis of the within-module degree (*z*) and participation coefficient (*P*) (67), nodes were divided into four categories: module hubs (*z* > 2.5, *P* > 0.62), network hubs (*z* > 2.5, *P* ≤ 0.62), connectors (*z* ≤ 2.5, *P* > 0.62), and peripherals (*z* ≤ 2.5, *P* ≤ 0.62) (68, 69). The four categories of nodes are shown in a *z–P* parameter plot, which was generated using the R package *ggClusterNet*.

We extracted subnetworks from the entire network by preserving the OTUs that existed in each sample (36, 70). A set of network topological properties was calculated using the R package *igraph*, including the average degree, average distance, betweenness centrality, degree centrality, eigenvector centrality, density, and transitivity.

### Community assembly analyses

The combined β-nearest taxon index (βNTI) and the Bray-Curtis-based Raup-Crick metric (RC_bray_) were used to estimate the relative importance of five assembly processes (HeS, DL, DF, HD, and HoS) in shaping the vertical distribution patterns of bacterial and archaeal communities in sediment cores. The βNTI was calculated as follows: first, a bacterial (or archaeal) phylogenetic tree was constructed using FastTree (version 2.1.11) with 1,000 bootstrap replicates and subsequently converted to an ultrametric tree via the *chronopl* function of the R package *ape*. Second, the mean nearest taxon distance metric (βMNTD), which represents the phylogenetic distance between each OTU in one community and its closest relative in a second community (71), was calculated using the ultrametric tree and the community data. At last, the βNTI was calculated as the difference between the observed βMNTD and the mean of the null distribution of the βMNTD (after 999 randomizations) normalized by the standard deviation (16, 72). The βNTI values separated deterministic processes from stochastic processes, and stochastic processes were subsequently partitioned using the RC_bray_ values. The RC_bray_ was estimated by the degree to which compositional variation among communities differed from the null expectation resulting from randomly distributed Bray-Curtis values (16, 73). The βNTI and RC_bray_ were calculated using the R packages *picante* and *iCAMP* (74), and the dominant assembly processes were evaluated based on the criteria in Table 1 (16, 18, 73).

**TABLE 1:** Criteria for evaluating the dominant assembly processes (16, 18, 72)

Criteria	Dominant processes
βNTI > 2	Heterogeneous selection
βNTI < –2	Homogeneous selection
–2 < βNTI < 2, RC_bray_ > 0.95	Dispersal limitation
–2 < βNTI < 2, RC_bray_ <– 0.95	Homogenizing dispersal
–2 < βNTI < 2,−0.95 < RC_bray_ < 0.95	Ecological drift and diversification

Additionally, we constructed Sloan’s neutral community model (NCM) (75) to assess the contribution of stochastic processes to microbial community assembly in the three cores. The NCM suggests that individuals are randomly chosen to die and are immediately replaced by immigrants from the metacommunity (source pool) with a probability of *m* or by descendants of the local community with a probability of 1–*m* (75), where *m* is the migration rate. The predicted frequencies of occurrence of OTUs were plotted with their mean relative abundances. OTUs were divided into three zones: upper, neutral, and lower zones, indicating higher, the same, and lower frequency of the occurrence of OTUs, respectively, in comparison with the predictions (76, 77). The R code for NCM implementation is available from GitHub (https://github.com/Weidong-Chen-Microbial-Ecology/Stochastic-assembly-of-river-microeukaryotes) (76).

### Statistical analyses

For α-diversity estimation, the ACE richness, Shannon-Wiener index, and Simpson index were calculated using the R package *vegan* (version 2.6-3) (78). For β-diversity estimation, the OTU table was square root-transformed, and a Bray-Curtis dissimilarity was calculated using the package *vegan*. Rare OTUs that were detected in fewer than 10% of samples were discarded for the following analyses (79). The dissimilarity among microbial communities was visualized by performing nonmetric multidimensional scaling analysis (NMDS) based upon Bray-Curtis dissimilarities (80), and the package *vegan* was used. Samples were divided into different groups based on the NMDS results. The difference in dispersion among sample groups was evaluated using the “betadisper” function (81), and significance was calculated using the “permutest” function (both functions are from the package *vegan*). Distance-based redundancy analysis (db-RDA) (82) was conducted using the package *vegan* to relate microbial communities with environmental variables and depth. Prior to db-RDA, three essential preprocessing steps were taken to ensure accurate results: (i) the relative abundances of OTUs were Hellinger-transformed, and environmental variables were used in log concentrations; (ii) the significance of all explanatory variables was examined using the envfit function in the package *vegan*, and only the significant ones were retained; and (iii) variables were manually selected on the basis of the variance inflation factor (VIF) until all the VIFs were below the recommended threshold of 10. Subsequent ANOVA test was conducted to assess the significance of the db-RDA results. Environmental variables were classified according to their common properties, and an RDA-based variation partitioning analysis (VPA) was performed using the package *vegan* to assess the percentage of community variation accounted for by each category of environmental variables. The VPA results were shown in a Venn diagram.

The correlation between subnetwork properties and environmental variables was assessed by the Mantel test and Pearson correlation. Box plots were used to visualize the differences among groups, and the statistical significances of pairwise comparisons were assessed using ANOVA followed by Tukey’s HSD test.

## RESULTS

### Physicochemical properties of sediment cores

The sediment physicochemical properties in the seven cores are provided in Table S2. The grain size composition was dominated by silt (mean 83.74%), with a small portion of clay (mean 13.56%). The contents of OC and ON were very low, and their mean values were 0.18% and 0.09%, respectively. The mean concentrations of major elements ranked as Fe_2_O_3_, MgO, K_2_O, MnO, CaO, Na_2_O, Al_2_O_3_, and P_2_O_5_, and the mean concentrations of minor elements were in the order of Cu > V > Sr > Ni > Cr > Zn > Co > Pb > Mo > As > Cd. Clay and organic matter (OC and ON) were more abundant in the seamount sediment cores, whereas trace elements such as As, Co, and Mo were significantly higher in the cores from the submarine valley (Fig. S1; Table S2).

### Microbial communities in sediment cores and their correlations with environmental factors

**Fig 2:**
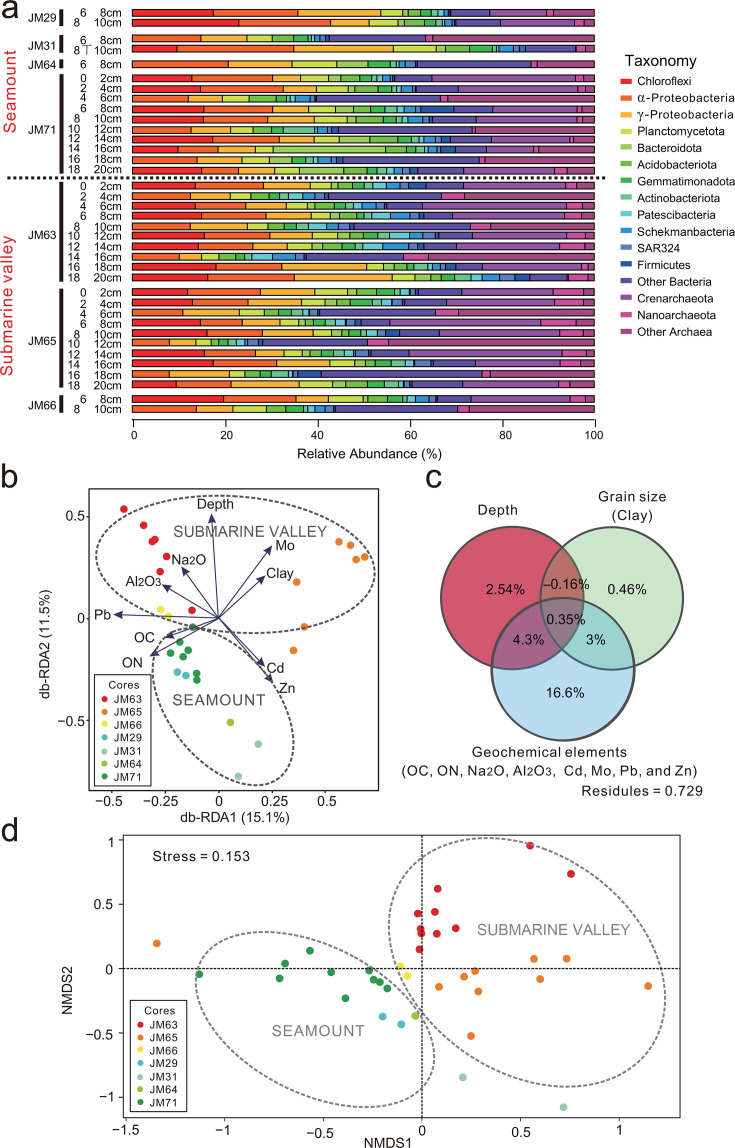
Microbial community composition and their relationships with environmental variables. (**a**) Vertical distribution of the microbial community composition in seven sediment cores. (**b**) The results of distance-based redundancy analysis (db-RDA) of samples and environmental variables. (**c**) Venn diagram showing the percentage of community variation explained by the three environmental variable categories. (**d**) Nonmetric multidimensional scaling analysis (NMDS) plot of microbial community structure (Bray-Curtis dissimilarities). In the db-RDA ordination plot, vectors represent the effect of each environmental variable on the two axes, length represents the relative size of the effect, and direction represents the correlation with respect to the two axes. OC, organic carbon; ON, organic nitrogen.

A total of 4,577 high-quality sequences were obtained, and they were identified into 3,105 bacterial and 334 archaeal OTUs at 97% similarity (Table S3). In all sediment cores, Chloroflexi, α-Proteobacteria, γ-Proteobacteria, Planctomycetota, Bacteroidota, Acidobacteriota, and Gemmatimonadota dominated bacterial communities at the phylum or subphylum level, and Thaumarchaeota (mainly Nitrososphaeria) and Nanoarchaeota (Woesearchaeales) dominated archaeal communities (Fig. 2a). The average total percentages of bacteria and archaea were 74.7% and 25.3%, respectively.

Results of db-RDA revealed that the first two axes explained 26.6% of the variation in microbial community structure. Figure 2b indicates the relationship between environmental variables and microbial community structure. Among all the variables, depth, clay, OC, ON, Al_2_O_3_, Na_2_O, Cd, Mo, Pb, and Zn significantly influenced microbial community structure (Table S4). The environmental factors that influenced the microbial communities differed among the seven cores, and the difference seemed to be related to the geographic locations. Cores JM63, JM65, and JM66 were located in the submarine valley, and the microbial communities were mainly positively correlated with depth, major elements (NaO and Al_2_O_3_), and clay. Cores JM29, JM31, JM64, and JM71 were located on the seamounts, and the microbial communities were mainly positively correlated with organic matter (OC and ON) and trace elements (Cd and Zn). The VPA results showed that variations in microbial community structure were primarily (16.6%) attributed to geochemical elements (OC, ON, NaO, Al_2_O_3_, Cd, Mo, Pb, and Zn), followed by the joint effects of three categories of environmental factors (7.49%), whereas a small proportion (2.54%) was caused by depth (Fig. 2c). Grain size (clay) alone contributed little to the variations in microbial community structure. The R^2^ and adjusted R^2^ of different groups of environmental variables for community compositional differences were shown in Table S4. The spatial distribution of microbial communities was determined by submarine topography, resulting in two clusters: seamount and submarine valley communities (Fig. 2b). This finding was reinforced by the NMDS results (Fig. 2d), in which all the communities were analyzed. The test of multivariate homogeneity of group dispersions resulted in a significantly lower dispersion of seamount communities compared with the submarine valley communities (average distance to centroid within group: seamount = 0.264, submarine valley = 0.324, Pr (>F) = 0.014).

**Fig 3:**
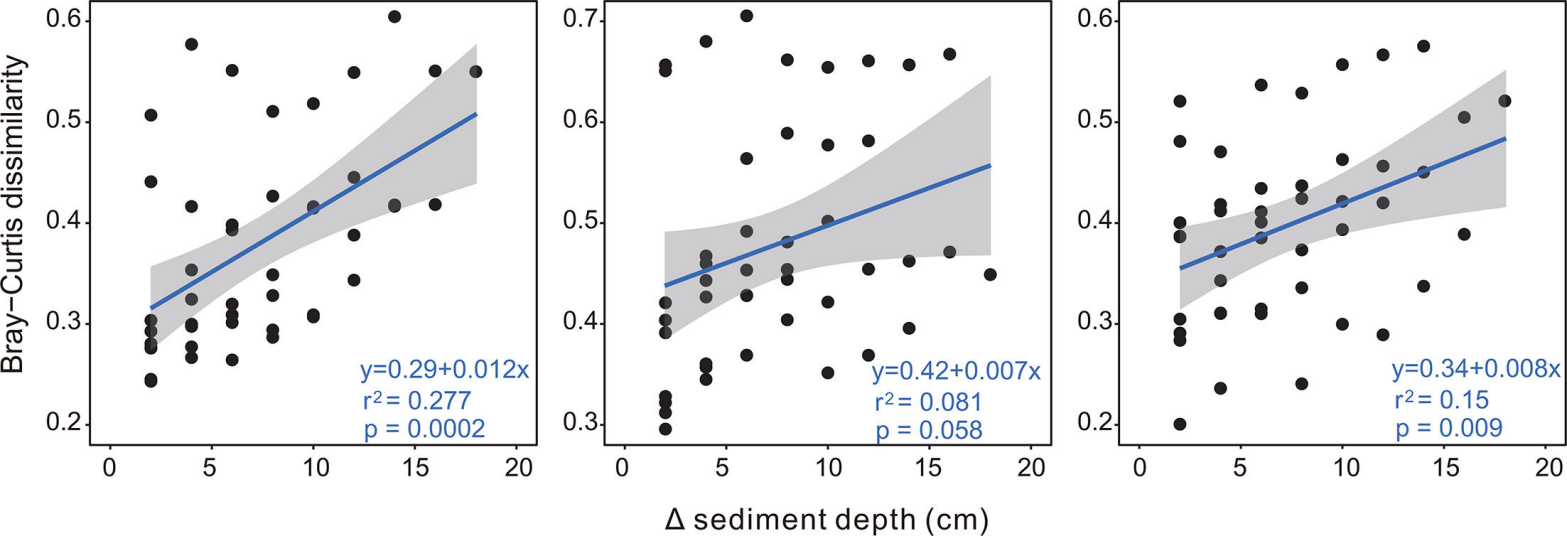
Vertical decay relationships of microbial communities in cores JM63, JM65, and JM71.

The vertical decay relationship (VDR) is defined as a decrease in community similarity with increasing sediment depth. The microbial communities showed a weak VDR in cores JM63 (slope = 0.012, r^2^ = 0.277, *P* = 0.0002) and JM71 (slope = 0.007, r^2^ = 0.15, *P* = 0.009), whereas no significant VDR was observed in core JM65 (slope = 0.008, r^2^ = 0.081, *P* = 0.058) (Fig. 3).

### Microbial co-occurrence patterns in sediment cores

**Fig 4:**
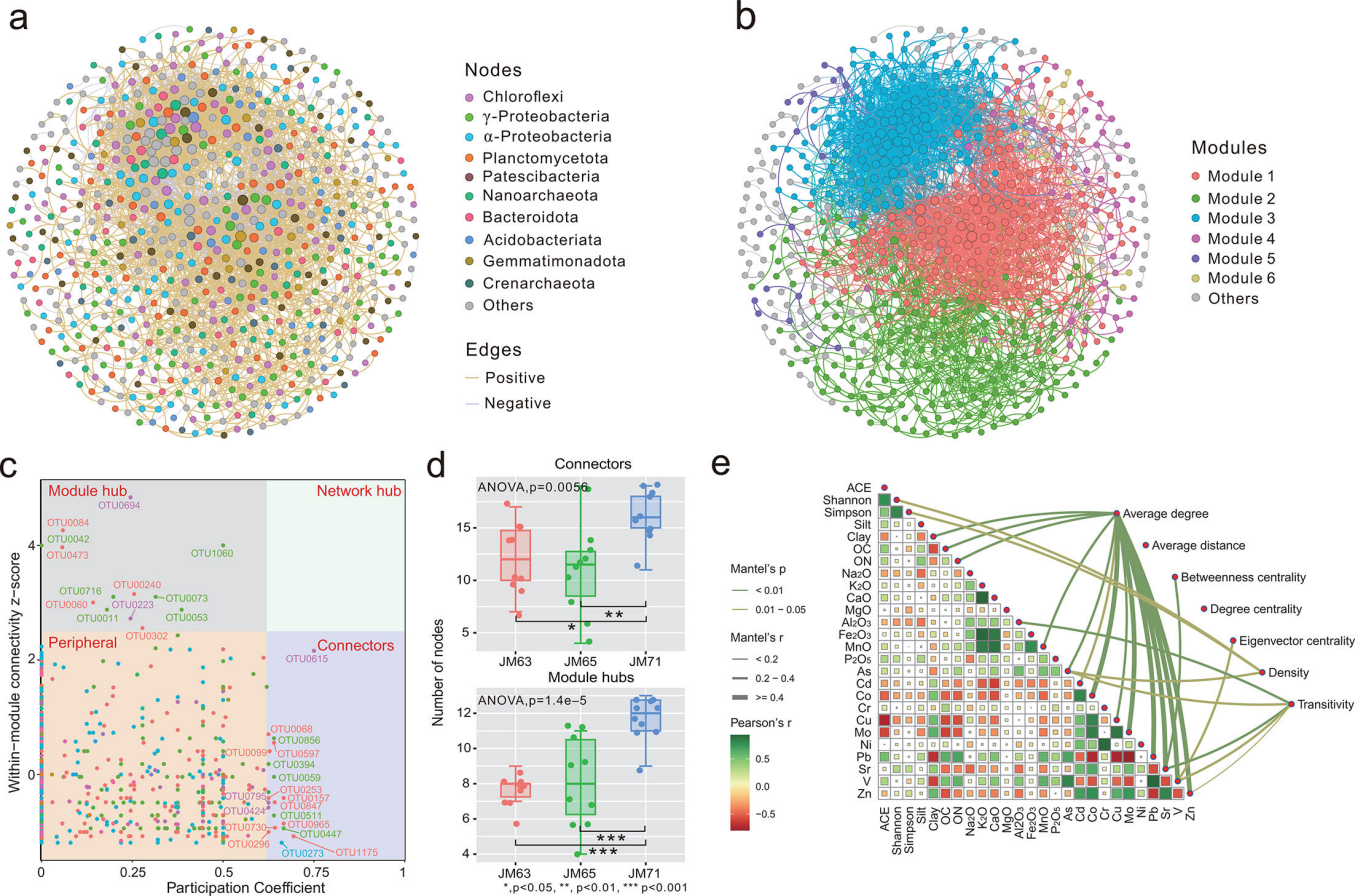
Co-occurrence network, modules, and topological properties. (**a**) Nodes in the network are colored by phylum, and the sizes are proportional to their degrees. Positive and negative correlations are denoted by different colors. (**b**) The network is divided into six large modules and several small modules. Each module is colored with different colors. (**c**) Nodes are classified as module hubs, network hubs, peripherals, and connectors in the z-*P* parameter space. (**d**) The number of connectors and the number of module hubs differ across the cores. Asterisks indicate the significance level of the difference (*P < 0.05, **P < 0.01, ***P < 0.001) as determined by Tukey’s HSD test. (**e**) The results of Pearson’s correlation analysis versus the Mantel test. The heatmap shows the Pearson correlations among environmental variables and α-diversities. The curves on the right side show the Mantel test results for the topological properties of the subnetworks and environmental variables. The width of each curve represents Mantel’s r statistic, and the color indicates a statistically significant value (*P*). OC, organic carbon; ON, organic nitrogen.

Network analyses were performed on cores JM63 (submarine valley), JM65 (submarine valley), and JM71 (seamount) because only these three cores contained complete vertical profiles of microbial communities. The microbial co-occurrence network (Fig. 4a) contained 684 nodes and 3,863 edges (2,838 positive and 1,025 negative), with an average degree (the average number of connections for each node in a network) of 11.3. The network consisted predominantly of 10 phyla: Chloroflexi (accounting for 11.8% of all nodes), γ-Proteobacteria (11.5%), α-Proteobacteria (11.4%), Planctomycetota (10.8%), Patescibacteria (6.9%), Nanoarchaeota (6.7%), Bacteroidota (5.6%), Acidobacteriota (5.3%), Gemmatimonadota (3.1%), and Thaumarchaeota (3.1%). In these phyla, the proportions of positive connections were similar to those of negative connections (Table S5).

The network was divided into six large modules and several small ones (Fig. 4b). The proportions of the main phyla in the six modules were similar to their proportions in the entire network (Table S6). The majority of OTUs (95%) were classified as peripherals, 1.9% as module hubs, and 3.1% as connectors (Fig. 4c). Module hubs were dominated by γ-Proteobacteria and Thaumarchaeota, whereas connectors comprised various phyla. Among the six modules, modules 1 and 2 had the highest numbers of module hubs and connectors (Table S7). The numbers of module hubs and connectors varied significantly among different cores (ANOVA, *P* < 0.01), and their highest numbers were both observed in core JM71 (Tukey’s HSD, *P* < 0.05 for each, Fig. 4d). However, the numbers of module hubs and connectors did not differ significantly between the other two cores (Tukey’s HSD, *P* > 0.05).

Subnetworks were extracted for each sample, and their topological properties were calculated. All the topological properties except eigenvector centrality varied significantly among the different cores among the three sediment cores (ANOVA, *P* < 0.05), and all the highest values were observed in core JM63 (Tukey’s HSD, *P* < 0.05 for each, Fig. S2). Most properties varied insignificantly between cores JM65 and JM71 (Tukey’s HSD, *P* > 0.05), with two exceptions: average degree was significantly higher in core JM71 (Tukey’s HSD, *P* < 0.001), whereas average distance was significantly higher in core JM65 (Tukey’s HSD, *P* = 0.046). The Mantel test revealed that average degree was significantly correlated with clay, organic matter (OC and ON), and most trace elements, and transitivity and betweenness centrality were mainly correlated with trace elements such as As, Sr, V, and Pb (Mantel’s r > 0.2, *P* < 0.05 for each) (Fig. 4e). Density exhibited a correlation with the α-diversities (Shannon-Wiener and Simpson indices). Average distance and degree centrality had no correlation with the environmental variables and α-diversities (*P* > 0.05).

### Microbial community assembly in sediment cores

**Fig 5:**
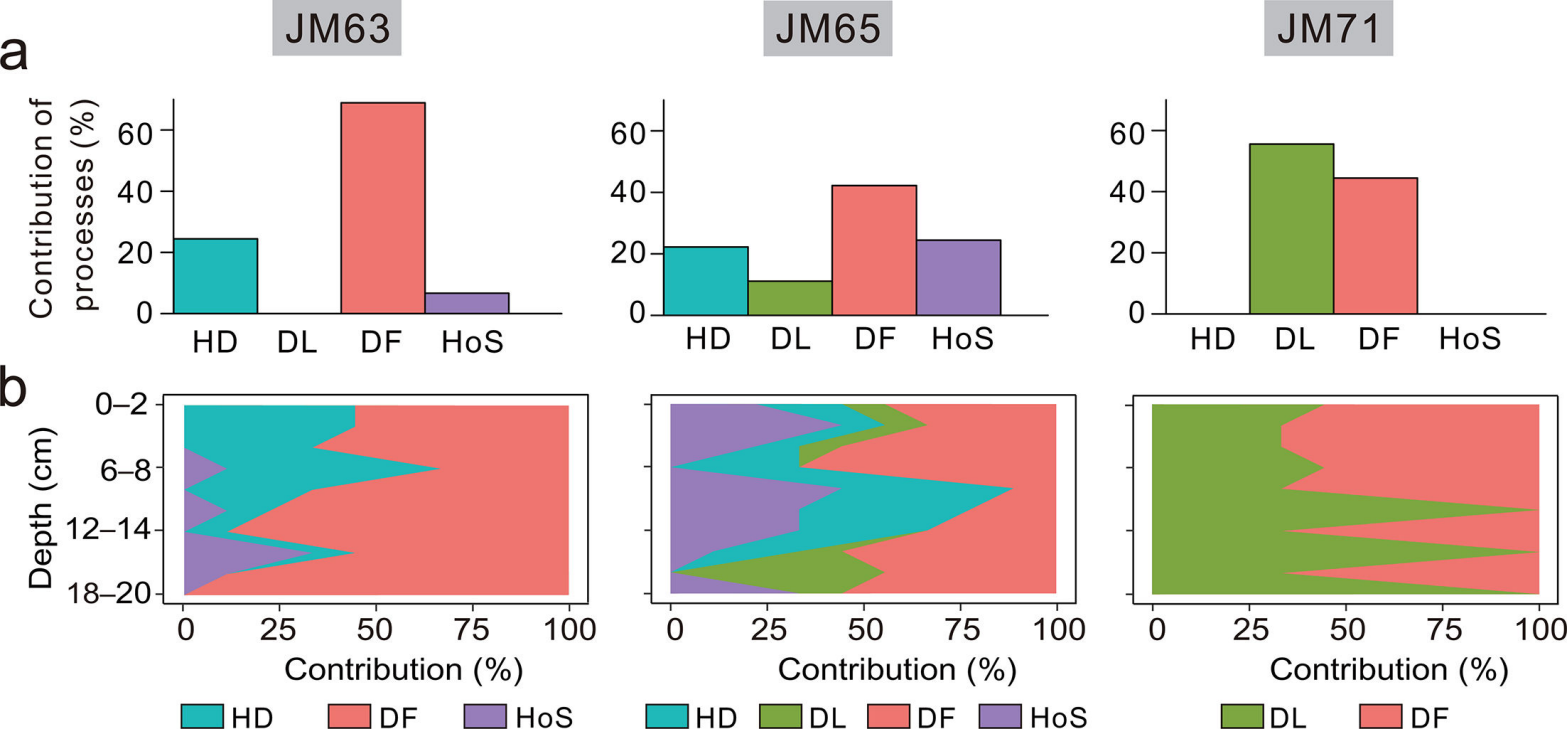
Overall contributions (**a**) and vertical variations of the relative contributions (**b**) of community assembly processes in cores JM63, JM65, and JM71. HoS, homogeneous selection; DL, dispersal limitation; HD, homogenizing dispersal; DF, drift.

The combined βNTI and RC_bray_ showed that HD, DL, DF, and HoS dominated the bacterial community assembly in three cores, JM63, JM65, and JM71, whereas DF contributed nearly 100% to the archaeal community assembly in these cores. For bacterial communities, the relative importance of each assembly process varied among different cores (Fig. 5a). In core JM63, DF played the most important role (68.9%) in shaping bacterial communities, HD was secondary (24.4%), and then HoS (6.7%). In core JM65, DF, HoS, and HD were the main assembly processes (42.2%, 24.2%, and 22.2%, respectively), whereas DL was less important (11.1%). In core JM71, bacterial community composition was jointly determined by DL and DF (55.6% and 44.4%, respectively). In each core, the relative importance of assembly processes varied with sediment depth (Fig. 5b). For example, in core JM63, DF increased with decreasing DL from the upper layer (0–10 cm) to the lower layer (10–20 cm). Core JM71 showed a transition from DF-dominated upper layers to DL-dominated lower layers. However, the relative contribution of DL and DF varied irregularly in core JM65.

**Fig 6:**
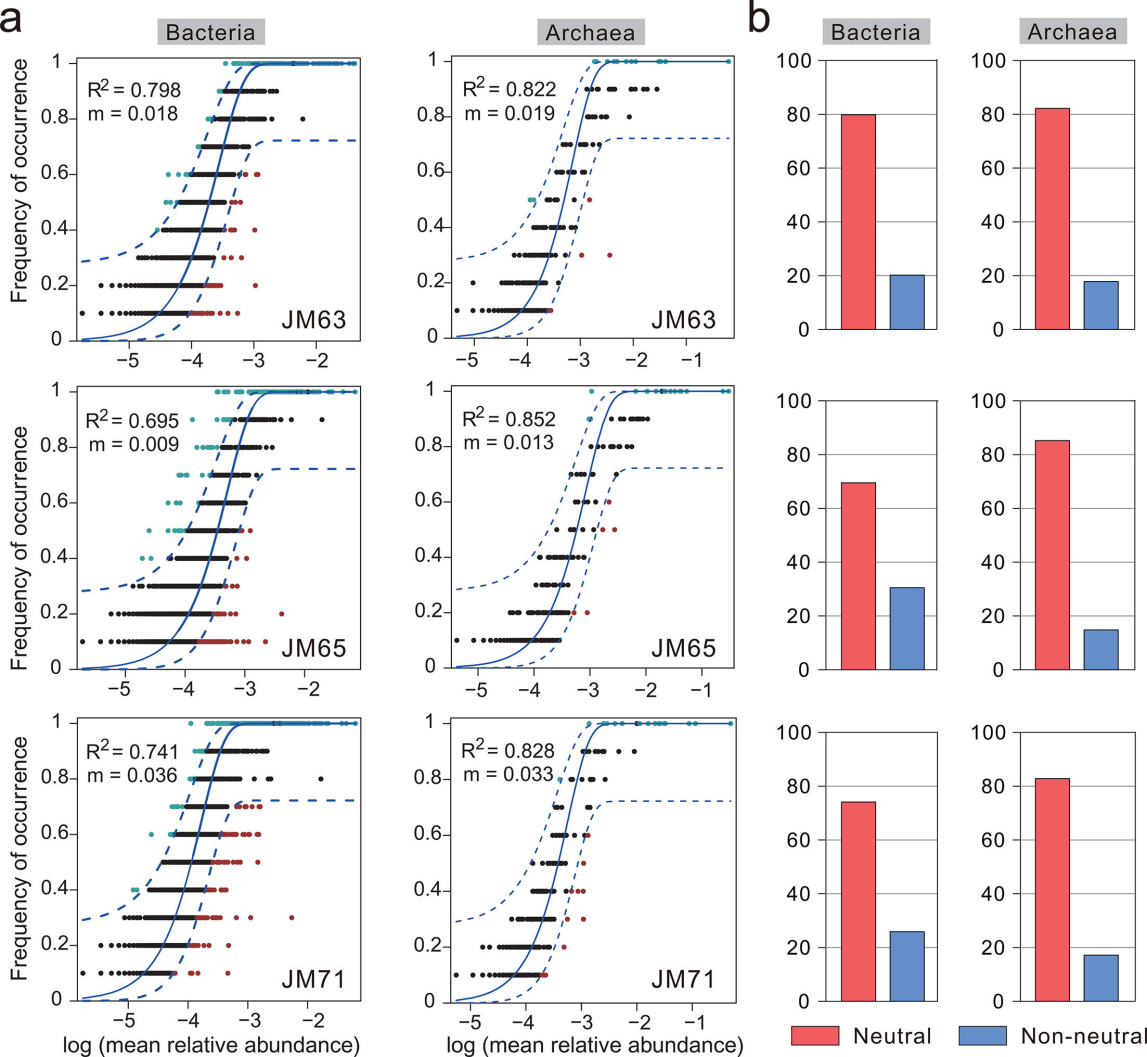
Fit of Sloan’s neutral community model of bacterial and archaeal communities (**a**) and percentages of community variance explained by neutral and non-neutral processes (**b**). In panel a, the solid line represents the best fit to the neutral model, and the two dashed lines represent the 95% confidence intervals of the predictions. The predictions of OTUs fall into the above, neutral, and below zones. R^2^ indicates the overall fit of the model, and *m* indicates the estimated migration rate.

The NCM showed that both bacterial and archaeal communities were driven predominantly by stochastic processes in the three cores (Fig. 6a and b). The R^2^ value of bacterial communities ranged from 0.695 to 0.741, reaching the highest value in core JM63. The R^2^ value of archaeal communities ranged from 0.822 to 0.852 with the highest value in JM65. The R^2^ value of bacterial communities was lower than that of archaeal communities in all cores. By comparison, the estimated migration rate (*m*) varied slightly between bacterial and archaeal communities, and both had the highest value in core JM71.

### Relationships between β-diversity and community assembly processes

**Fig 7:**
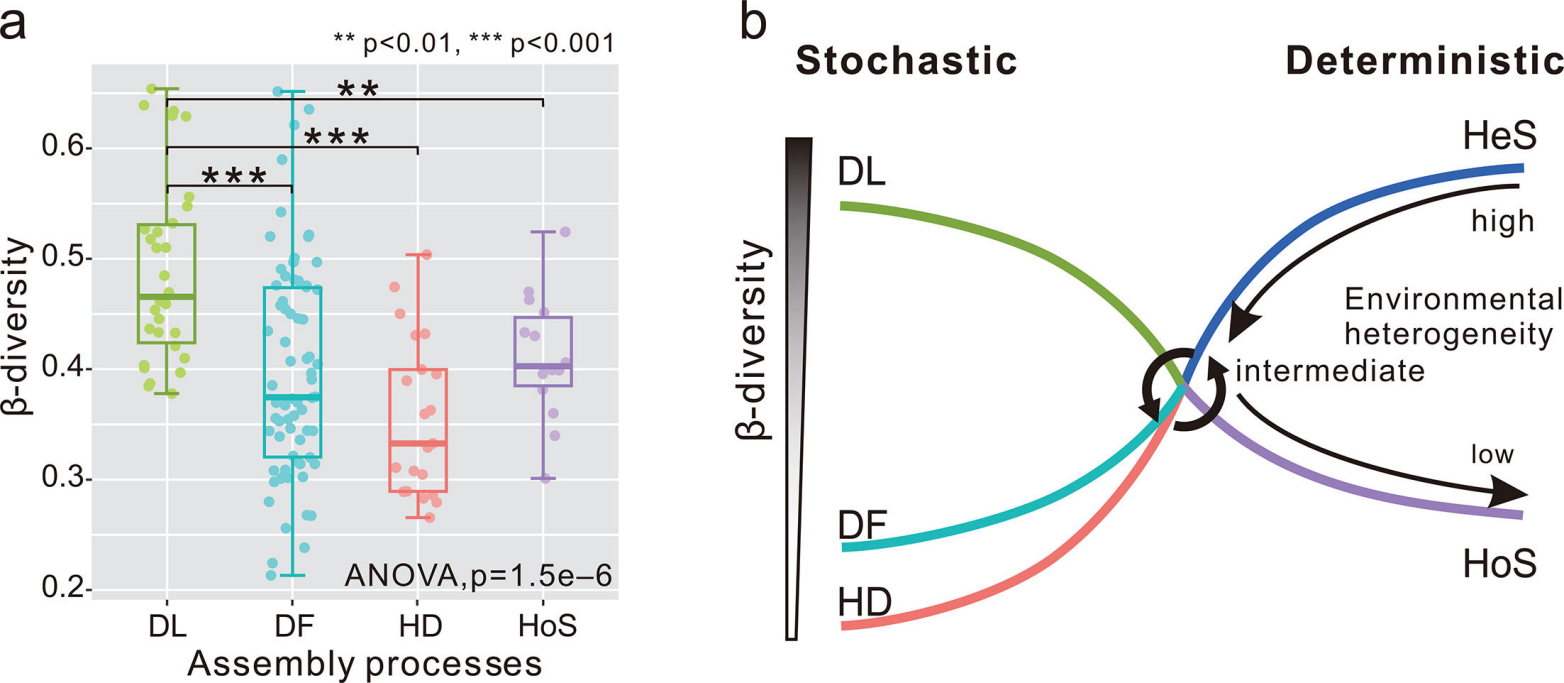
Relationship between β-diversity and community assembly processes and the conceptual model. (**a**) Box plots showing differences in β-diversity among local communities driven by different assembly processes on the basis of the bacterial community data. Asterisks indicate significance: **P < 0.01 and ***P < 0.001 based on Tukey’s HSD test. (**b**) The conceptual model shows how changes in dominant assembly processes lead to changes in β-diversity. In this model, environmental heterogeneity determines which process dominates community assembly. With decreasing environmental heterogeneity, the dominant assembly process changes from HeS to stochastic processes such as DL, DF, and HD, and then to HoS. HoS, homogeneous selection; HeS, heterogeneous selection; DL, dispersal limitation; HD, homogenizing dispersal; DF, drift.

To reveal how β-diversity changes with different assembly processes, we compared the difference in bacterial β-diversity among communities driven by different assembly processes. The results showed that bacterial β-diversity varied significantly among the four processes: DL, DF, HD, and HoS (ANOVA, *P* < 0.001). Specifically, β-diversity was significantly greater when the community assembly was dominated by DL than by HD, DF, or HoS (Tukey’s HSD, *P* < 0.05) (Fig. 7a). It can be inferred that DL caused an increase in β-diversity, whereas HD, DF, and HoS caused a decrease in β-diversity. Additionally, HoS resulted in a slightly higher β-diversity than DF and DF resulted in a slightly higher β-diversity than HD; however, the differences among the three processes were not statistically significant (Tukey’s HSD, *P* > 0.05). Since DF was the only process that shaped archaeal community assembly, the relationship between archaeal β-diversity and community assembly processes was largely unknown.

## DISCUSSION

The results of this study partly support the hypothesis that β-diversity is enhanced by HeS and DL but decreased by HD, DF, and HoS at local scales. Since HeS did not contribute to the community assembly process, the comparison of the impact of HeS with those of the other processes needs further study. In our hypothesis, β-diversity was calculated on the basis of the Bray-Curtis distance, which is widely used in community ecology (51, 83–85), whereas other measures of β-diversity (86, 87) are outside the scope of this study. Ferrenberg et al. (88) provided another piece of evidence to support this hypothesis that after wildfire disturbance, soil bacterial communities experience two stages of increasing β-diversity, initially caused by dispersal and then by niche-based processes (i.e., selection). The mechanism driving the variation in β-diversity is as follows. If environmental conditions change through space (i.e., heterogeneous environment), the process of selection differentiates microbial community composition among locations by selecting taxa that are relatively better adapted to local conditions and excluding those that are not adapted (45, 89). Similarly, if dispersal is limited, the movement of species from one location to another will be restricted, resulting in a great difference in community composition between the locations (18). In contrast, other factors, such as homogeneous environments, high dispersal rates, and stochastic processes of growth and death, tend to homogenize community composition (18, 90), leading to low β-diversity.

Huber et al. (45) proposed a conceptual model to explain how changes in environmental heterogeneity lead to changes in assembly processes and ultimately cause changes in β-diversity. We improved this model by clarifying the different effects among stochastic processes such as HD, DL, and DF on β-diversity (Fig. 7b). In a system with high environmental heterogeneity, HeS is the most important process, resulting in a high β-diversity. At intermediate values of environmental heterogeneity, stochastic processes overwhelm the effects of selection. If DL dominates assembly processes, β-diversity does not substantially change. However, if HD or DF prevails, β-diversity decreases significantly. While in a system with low environmental heterogeneity, community assembly is driven mainly by HoS, whereas stochastic processes are less important. The variance of β-diversity depends on which kind of processes dominates community assembly at intermediate levels of environmental heterogeneity. Apparently, a transition from DL to HoS leads to a significant decrease in β-diversity. Changes among HD, DF, and HoS may lead to a slight increase in β-diversity in the sequence HD <DF < HoS. Therefore, changes in the relative contributions of stochastic and deterministic processes cannot simply determine the directions of β-diversity variation, and all five assembly processes should be analyzed in detail, if possible. Here, we use the modified model to explain the shifts of bacterial β-diversity in the three sediment cores. The main causes of increasing β-diversity in core JM63 were the increasing contribution of DF and the declining contribution of HD. A transition from the dominance of DF to that of DL was the crucial factor for the increase of β-diversity in core JM71. However, assembly processes varied irregularly in core JM65, and other factors such as species interactions were likely to affect β-diversity, for example, stronger interactions cause low β-diversity, whereas weaker interactions cause high β-diversity (91).

To disentangle the effects of each assembly process on β-diversity, we proposed a new method in which the differences in β-diversity among communities driven by different assembly processes were compared. Previous studies have usually partitioned community variation into environmental and spatial components to distinguish the influences of environmental selection and dispersal on β-diversity (41, 53, 84, 89, 92–94). If community variation is primarily explained by environmental variation, selection is likely the determinant of β-diversity, whereas if community variation is primarily explained by spatial variation, dispersal likely plays a stronger role in determining β-diversity (84, 95). However, predicting the magnitude and direction of changes in β-diversity caused by selection or dispersal is impossible. Alternatively, our approach quantified the relative influence of each assembly process on β-diversity, and how a transition from one process to another altered β-diversity. From this perspective, our approach worked better than those used in previous studies.

Unlike previous studies that have focused on microbial community assembly in surface sediments on seamounts (10, 14), this study contributes to microbial community assembly in seamount sediment cores. The βNTI-based null model showed that bacterial community assembly was dominated by DF, DL, HD, and HoS, and their relative importance varied across different cores. DL was one of the dominant processes in core JM71, but it played a relatively small role in cores JM63 and JM65. HD was important in cores JM63 and JM65, but it was absent in core JM71. The assembly of bacterial communities seemed to be related to the geographic locations: seamount versus submarine valley. Previous studies have found that the relative importance of assembly processes changes with water depth (22). Accordingly, we thought that water depth may be the main controlling factor for the variations in the relative importance of DL and HD, for example, DL showed a decreasing trend, while HD showed an increasing trend with increasing water depth. However, archaeal community assembly was totally dominated by DF in the three cores. The dominant community assembly mechanism differed between bacteria and archaea, and the possible reason is due to the small sizes of the archaeal communities since drift can act alone in small communities through probabilistic factors (48).

The NCM was used to assess the contribution of neutral processes in microbial community assembly. The total fit of the NCM is shown by the R^2^ values, and higher R^2^ values denote a better fit of the NCM of community assembly (96), indicating that the neutral (stochastic) processes have a greater role in community assembly (97). We compared the R^2^ values with the proportions of stochastic processes as indicated by the βNTI indices. The R^2^ values were higher for greater proportions of stochastic processes, suggesting that the NCM results were comparable with the βNTI analyses. Isabwe et al. (97) assumed that the R^2^ values of the NCM have strong negative or positive correlations with the relative contribution of certain assembly processes. However, only three systems (sediment cores) were analyzed in this study, and the above correlations were still largely unresolved.

The microbial co-occurrence networks were compared among cores JM63, JM65, and JM71, and both node-level and network-level properties were used. Keystone taxa are crucial for maintaining microbial community structure and functioning (29). In this study, the connectors and module hubs identified in the z-*P* parameter space (Fig. 4c) were chosen as potential keystone taxa. Connectors are nodes that have more connections with others between modules, and module hubs are nodes that have more connections with others within a module (69, 98), and their removal is harmful to network connectivity (99). We propose that the more abundant the keystone taxa are, the less likely they are to be removed. We can infer that the microbial networks in core JM71 were the most stable because these networks had the highest numbers of keystone taxa. Microbial networks are unstable when under environmental stress (26), but the mechanism remains unknown. In this study, microbial communities were less influenced by selection in core JM71 than those in the other two cores. More keystone taxa were likely maintained in core JM71 under weaker selection, promoting the stability of the microbial network; ‌however, in the other cores‌, the higher selection pressure may cause the decline in the number of keystone taxa, resulting in the destabilization of microbial networks. At the network level, a set of topological properties was used to evaluate the network stability in the three cores. The networks in core JM71 had the highest average degree and the lowest average distance, and according to Jiao et al. (96), microbial taxa were more interconnected, and their relationships were closer in this core than in the other cores. This is in agreement with the fact that this core contained the most stable networks, as mentioned above. However, network robustness analysis was not conducted in this study, and this conclusion was incomplete.

The network properties were associated with the environmental variables, and the results showed that the properties were affected by certain variables, among which trace elements such as As, Sr, V, and Pb were the strongest. These elements are harmful to microbial communities, which may be the main driving force for the variability of microbial co-occurrence patterns among the cores.

This study investigated the influences of submarine topography and sediment physicochemical properties on microbial communities. The submarine topography controlled the distribution of the microbial communities and divided them into two clusters: seamount and submarine valley communities. The former were mainly positively affected by organic matter (OC and ON) and trace elements, whereas the latter were mainly positively affected by clay and major elements. The effect of organic matter on the seamount communities may be explained by the topographic effect. Seamount topography affects ocean currents and mixing, leading to high primary productivity in the upper water column (5, 100, 101). The enhancement of nutrients from the upper water column provides benefits for seamount benthic communities (11).

### Conclusions

Local-scale variation in β-diversity is driven by changes in local community assembly processes, and DL increases β-diversity, whereas HD and HoS decrease β-diversity. Although this conclusion is tested with seamount bacterial communities, it may also be applicable to other ecosystems. This study demonstrates that stochastic processes shape the vertical distribution patterns of microbial communities in the sediment cores and provides a case study of the assembly mechanism of microbial communities in subsurface sedimentary environments. In addition, this study suggests that the variability of microbial community composition and co-occurrence patterns in seamount sediments is driven mainly by trace elements, improving the understanding of microbial ecology in globally widespread seamounts.

## Data Availability

The raw reads were deposited in the Genome Sequence Archive (https://ngdc.cncb.ac.cn/gsa/) under accession number CRA025601.

## SUPPLEMENTAL MATERIAL

Fig. S1 aem.00732-26-s0001.pdf Comparison of the mean concentrations of physiochemical properties in different sediment cores.

Fig. S2 aem.00732-26-s0002.pdf Box plots showing differences in the network properties among cores JM63, JM65, and JM71.

Fig. S3 aem.00732-26-s0003.pdf Vertical variations of average β-diversity in cores JM63, JM65, and JM71.

Table S1 aem.00732-26-s0004.pdf Sample collection and samples used for DNA extraction.

Table S2 aem.00732-26-s0005.pdf Sediment physiochemical properties in seven cores (the upper table) and the differences in the physiochemical properties between the seamount and valley sediment cores (the lower table).

Table S3 aem.00732-26-s0006.xlsx The relative abundances of microbial OTUs in the seamount sediments and taxonomy assignments.

Table S4 aem.00732-26-s0007.pdf The significance test of the db-RDA results (the upper table) and the R2 and adjusted R2 of different groups of environmental variables for community compositional differences (the lower table).

Table S5 aem.00732-26-s0008.pdf Percentages of positive and negative connections among different phyla.

Table S6 aem.00732-26-s0009.pdf Comparing the proportions of the main phyla (%) in the six large modules with those in the entire network.

Table S7 aem.00732-26-s0010.pdf Numbers of module hubs, connectors, and peripherals in the six large modules.

ASM does not own the copyrights to Supplemental Material that may be linked to, or accessed through, an article. The authors have granted ASM a non-exclusive, world-wide license to publish the Supplemental Material files. Please contact the corresponding author directly for reuse.
