# Supplementary material for: Microbial co-occurrence patterns and community assembly in seamount sediment cores: disentangling the effects of assembly processes on β-diversity
Source: Appl Environ Microbiol. 2026 Jun 18;92(7):e00732-26. doi: 10.1128/aem.00732-26 (PMC13390388; doi:10.1128/aem.00732-26)
Supplement: Fig. S1 — Comparison of the mean concentrations of physiochemical properties in different sediment cores. [file aem.00732-26-s0001.pdf]

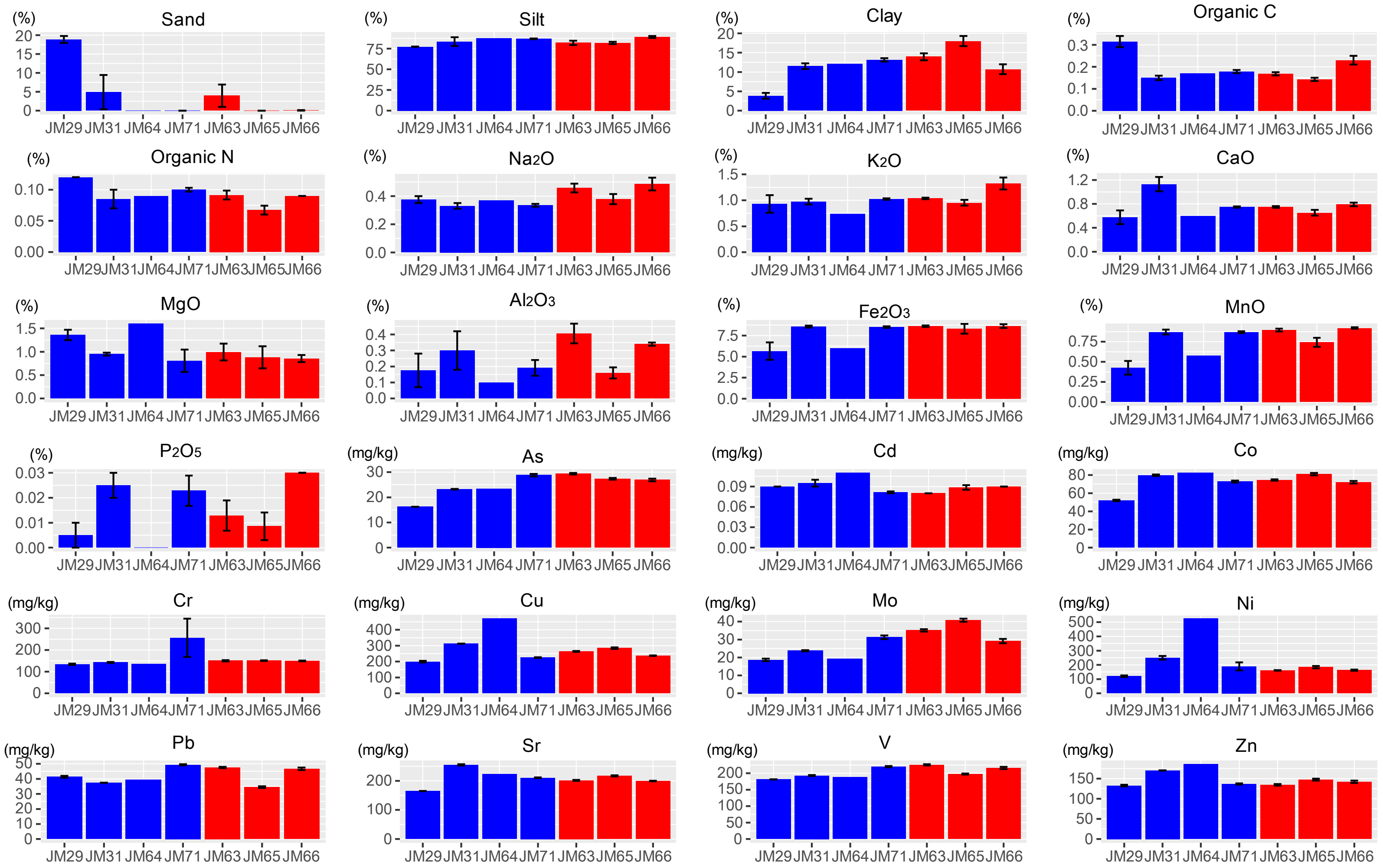

FIG S1 Comparison of the mean concentrations of physiochemical properties in different sediment cores. Blue and red bars denote sediment cores that collected from the seamount and submarine valley, respectively. Black lines denote standard deviations.
