# Supplementary material for: Microbial co-occurrence patterns and community assembly in seamount sediment cores: disentangling the effects of assembly processes on β-diversity
Source: Appl Environ Microbiol. 2026 Jun 18;92(7):e00732-26. doi: 10.1128/aem.00732-26 (PMC13390388; doi:10.1128/aem.00732-26)
Supplement: Fig. S2 — Box plots showing differences in the network properties among cores JM63, JM65, and JM71. [file aem.00732-26-s0002.pdf]

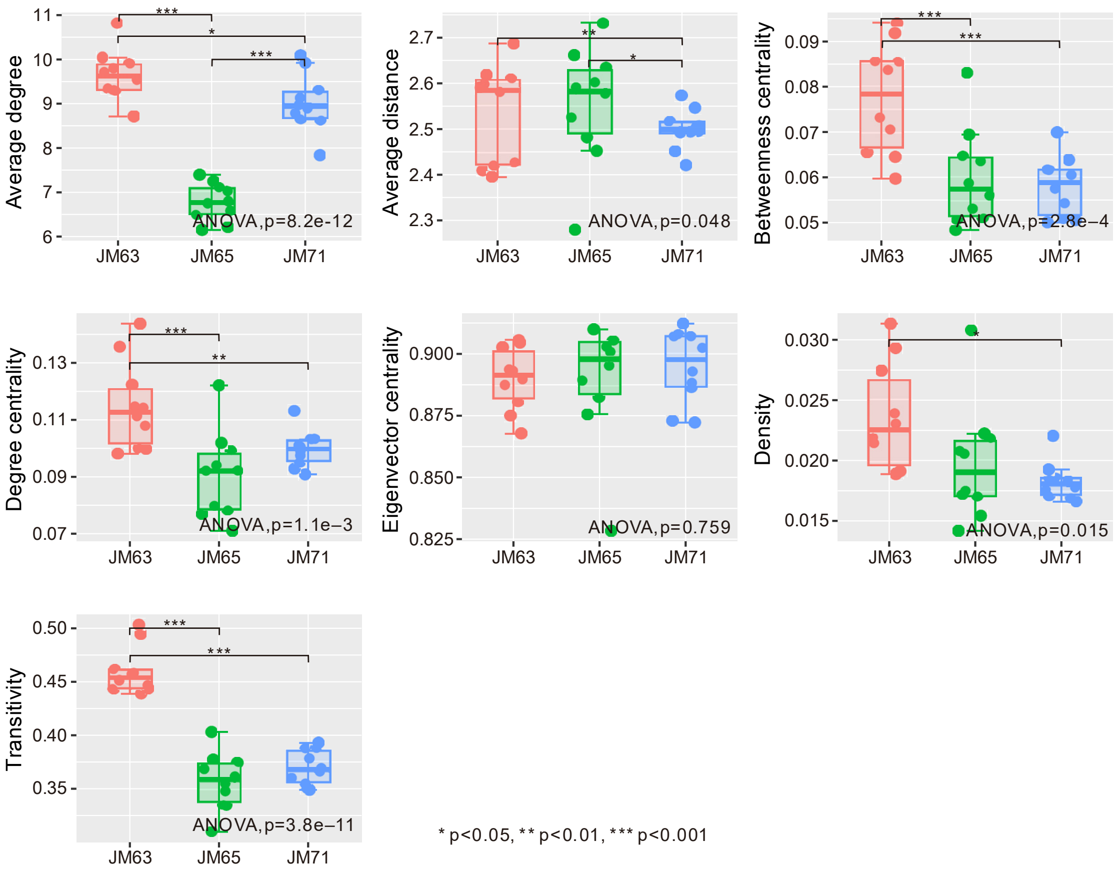

FIG S2 Box plots showing differences in sub-network properties (average degree, average distance, betweeness centrality, degree centrality, eigenvector centrality, density, and transitivity) among cores JM63, JM65, and JM71. Asterisks indicate significance: *p<0.05, **p < 0.01, and ***p < 0.001 based on Tukey's HSD test.
