# Supplementary figures and images for: Microbial co-occurrence patterns and community assembly in seamount sediment cores: disentangling the effects of assembly processes on β-diversity
Source: Appl Environ Microbiol. 2026 Jun 18;92(7):e00732-26. doi: 10.1128/aem.00732-26 (PMC13390388; doi:10.1128/aem.00732-26)

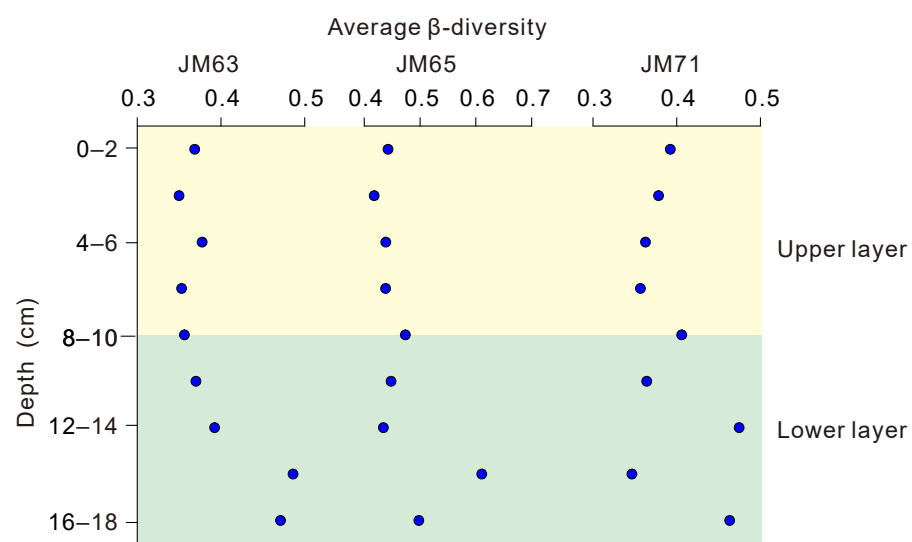

FIG S3 Vertical variations of average  $\beta$ -diversity in cores JM63, JM65, and JM71.

Supplement: Fig. S3 — Vertical variations of average β-diversity in cores JM63, JM65, and JM71. [file aem.00732-26-s0003.pdf]
