# Supplementary material for: Microbial co-occurrence patterns and community assembly in seamount sediment cores: disentangling the effects of assembly processes on β-diversity
Source: Appl Environ Microbiol. 2026 Jun 18;92(7):e00732-26. doi: 10.1128/aem.00732-26 (PMC13390388; doi:10.1128/aem.00732-26)
Supplement: Table S1 — Sample collection and samples used for DNA extraction. [file aem.00732-26-s0004.pdf]

Table S1 Sample collection and samples used for DNA extraction.

Core ID	Collecting date	Location	Water depth(m)	Sediment depth(cm)	Sample weight for DNA extraction	SUCCESS /FAILURE
JM29	2019.6.20	The bottom of the Guyu Seamount	5231	0–2	ALL(~10g)	FAILURE
				2–4	ALL(~10g)	FAILURE
				4–6	ALL(~10g)	FAILURE
				6–8	HALF(~5g)	SUCCESS
				8–10	HALF(~5g)	SUCCESS
				10–12	ALL(~10g)	FAILURE
				12–14	ALL(~10g)	FAILURE
				14–16	ALL(~10g)	FAILURE
				16–18	ALL(~10g)	FAILURE
				18–20	ALL(~10g)	FAILURE
JM31	2019.6.21	The summit of the Guyu Seamount	4448	0–2	ALL(~10g)	FAILURE
				2–4	ALL(~10g)	FAILURE
				4–6	ALL(~10g)	FAILURE
				6–8	HALF(~5g)	SUCCESS
				8–10	HALF(~5g)	SUCCESS
				10–12	ALL(~10g)	FAILURE
				12–14	ALL(~10g)	FAILURE
				14–16	ALL(~10g)	FAILURE
				16–18	ALL(~10g)	FAILURE
				18–20	ALL(~10g)	FAILURE
JM63	2020.8.11	Submarine valley	6368	0–2	ALL(~10g)	SUCCESS
				2–4	ALL(~10g)	SUCCESS
				4–6	ALL(~10g)	SUCCESS
				6–8	HALF(~5g)	SUCCESS
				8–10	HALF(~5g)	SUCCESS
				10–12	HALF(~5g)	SUCCESS
				12–14	HALF(~5g)	SUCCESS
				14–16	HALF(~5g)	SUCCESS
				16–18	HALF(~5g)	SUCCESS
				18–20	HALF(~5g)	SUCCESS
JM64	2020.8.12	The bottom of an unknown submarine mountain	5345	0–2	ALL(~10g)	FAILURE
				2–4	ALL(~10g)	FAILURE
				4–6	ALL(~10g)	FAILURE
				6–8	HALF(~5g)	SUCCESS
				8–10	ALL(~10g)	FAILURE
				10–12	ALL(~10g)	FAILURE
				12–14	ALL(~10g)	FAILURE
				14–16	ALL(~10g)	FAILURE
				16–18	ALL(~10g)	FAILURE
				18–20	ALL(~10g)	FAILURE

JM65	2020.8.13	Submarine valley	5997	0–2	ALL(~10g)	SUCCESS
				2–4	ALL(~10g)	SUCCESS
				4–6	ALL(~10g)	SUCCESS
				6–8	HALF(~5g)	SUCCESS
				8–10	HALF(~5g)	SUCCESS
				10–12	HALF(~5g)	SUCCESS
				12–14	HALF(~5g)	SUCCESS
				14–16	HALF(~5g)	SUCCESS
				16–18	HALF(~5g)	SUCCESS
				18–20	HALF(~5g)	SUCCESS
JM66	2020.8.13	Submarine valley	5999	0–2	ALL(~10g)	FAILURE
				2–4	ALL(~10g)	FAILURE
				4–6	ALL(~10g)	FAILURE
				6–8	HALF(~5g)	SUCCESS
				8–10	HALF(~5g)	SUCCESS
				10–12	ALL(~10g)	FAILURE
				12–14	ALL(~10g)	FAILURE
				14–16	ALL(~10g)	FAILURE
				16–18	ALL(~11g)	FAILURE
				18–20	ALL(~12g)	FAILURE
JM71	2020.8.14	The hillside of the Guyu Seamount	5386	0–2	ALL(~10g)	SUCCESS
				2–4	ALL(~10g)	SUCCESS
				4–6	ALL(~10g)	SUCCESS
				6–8	HALF(~5g)	SUCCESS
				8–10	HALF(~5g)	SUCCESS
				10–12	HALF(~5g)	SUCCESS
				12–14	HALF(~5g)	SUCCESS
				14–16	HALF(~5g)	SUCCESS
				16–18	HALF(~5g)	SUCCESS
				18–20	HALF(~5g)	SUCCESS
