# Supplementary material for: Microbial co-occurrence patterns and community assembly in seamount sediment cores: disentangling the effects of assembly processes on β-diversity
Source: Appl Environ Microbiol. 2026 Jun 18;92(7):e00732-26. doi: 10.1128/aem.00732-26 (PMC13390388; doi:10.1128/aem.00732-26)
Supplement: Table S2 — Sediment physiochemical properties in seven cores (the upper table) and the differences in the physiochemical properties between the seamount and valley sediment cores (the lower table). [file aem.00732-26-s0005.pdf]

Table S2 Sediment physiochemical properties in seven cores (the upper table) and the differences in the physiochemical properties between the seamount and valley sediment cores (the lower table).

Core	Depth	Sand	Silt	Clay	Org.C	Org.N	Na_2O	K_2O	CaO	MgO	Al_2O_3	Fe_2O_3	MnO	P_2O_5	As	Cd	Co	Cr	Cu	Mo	Ni	Pb	Sr	V	Zn
	(cm)	(%)													(mg/kg)										
JM29	6–8	17.93	77.48	4.59	0.34	0.12	0.4	1.1	0.69	1.25	0.28	6.7	0.51	0	16.25	0.09	51.27	137.16	204.93	19.36	126.44	40.82	164.45	181.47	134.63
	8–10	19.79	77.13	3.08	0.29	0.12	0.35	0.76	0.46	1.47	0.07	4.61	0.34	0.01	16.22	0.09	52.73	131.03	193.92	17.94	115.74	41.98	165.28	180.85	130.42
JM31	6–8	0.39	88.82	10.79	0.16	0.07	0.31	0.92	1.01	0.98	0.18	8.46	0.84	0.02	23.31	0.09	78.96	145.08	312.51	23.43	236.68	37.46	256.95	194.78	170.72
	8–10	9.44	78.33	12.23	0.14	0.1	0.35	1.03	1.25	0.92	0.42	8.68	0.9	0.03	23.05	0.1	80.54	140.54	312.67	24.08	262.17	37.36	252.47	191.52	169.94
JM63	6–8	0	86.15	13.85	0.2	0.07	0.59	1.01	0.73	0.92	0.18	8.41	0.86	0	28.56	0.08	72.41	148.8	265.8	32.92	166.56	46.56	196.85	221.28	131.01
	8–10	0.47	85.89	13.64	0.16	0.11	0.49	1.01	0.68	0	0.39	8.38	0.87	0	28.68	0.08	73.82	146.17	267.21	33.85	164.58	46.6	197.61	223.17	133.3
	10–12	0	81.23	18.77	0.16	0.1	0.44	1.07	0.76	1.18	0.38	8.74	0.89	0.03	28.7	0.08	72.87	145.45	255.54	34.3	152.21	47.04	198.08	222.48	132.86
	12–14	21.26	67.08	11.66	0.17	0.06	0.51	1.08	0.75	0.96	0.53	8.74	0.9	0	29.21	0.08	74.65	143.17	253.98	34.67	154.05	47.34	198.85	220.83	129
	14–16	1.02	84.9	14.09	0.19	0.1	0.4	1.07	0.79	1.45	0.47	8.75	0.94	0.03	30.24	0.08	78.34	152.04	269.6	35.96	167.56	49.21	206.57	232.83	134.55
	16–18	5	83.33	11.67	0.15	0.1	0.43	0.99	0.75	1.18	0.65	8.33	0.84	0	29.87	0.08	74.77	150.97	262.05	35.44	158.37	47.69	205.37	227.46	135.55
	18–20	0	86.08	13.92	0.15	0.1	0.34	1.03	0.78	1.27	0.24	8.95	0.97	0.03	30.47	0.08	74.86	166.53	273.75	38.54	165.07	48.8	206.18	231.31	145.73
JM64	6–8	0	87.87	12.13	0.17	0.09	0.37	0.74	0.6	1.6	0.1	6.01	0.58	0	23.43	0.11	82.27	135.86	470.55	19.28	525.11	39.49	222.82	187.88	185.67
JM65	6–8	0	83.88	16.12	0.15	0.08	0.22	0.67	0.38	1.55	0	5.31	0.48	0	27.42	0.1	86.44	157.05	297.64	40.02	210.01	36.54	217.33	202.46	151.08
	8–10	0	85.29	14.71	0.14	0.05	0.43	0.97	0.68	0	0.15	8.81	0.91	0.03	28.12	0.1	83.88	154.09	297.48	40.36	201.95	36.12	219.67	202.21	151.49
	10–12	0	81.48	18.52	0.13	0.09	0.41	1.1	0.75	1.05	0.18	9.69	0.82	0	25.94	0.08	78.36	145.14	270.91	38.22	167.66	33.35	209.24	190.56	140.48
	12–14	0	85.23	14.77	0.18	0.05	0.36	0.87	0.63	1.34	0.21	7.39	0.63	0	26.37	0.08	76.16	145.82	266.94	39.31	144.46	32.71	211.15	193.01	138.31
	14–16	0	75.55	24.45	0.14	0.05	0.52	1.08	0.73	1.14	0.29	9.45	0.86	0	27.57	0.09	80.89	152.36	285.81	40.02	189.61	33.93	218.52	197.21	151.46
	16–18	0	83.06	16.94	0.12	0.06	0.31	0.98	0.7	1.09	0.1	8.63	0.75	0.03	27.95	0.08	80.2	150.92	284.61	42.76	174.59	34.09	222.2	198.16	144.81
	18–20	0	79.54	20.46	0.14	0.09	0.4	1	0.69	0	0.19	8.86	0.75	0	28.03	0.09	81.29	152.9	289.49	44.95	199.15	35.09	220.82	199.55	154.86
JM66	6–8	0	88.02	11.99	0.25	0.09	0.44	1.21	0.82	0.93	0.33	8.85	0.91	0.03	26.37	0.09	70.69	148.4	235.58	27.91	158.32	45.86	198.61	212.77	144.96

	8−10	0.16	90.41	9.43	0.21	0.09	0.53	1.44	0.76	0.78	0.35	8.4	0.93	0.03	27.42	0.09	73.44	151.27	238.71	30.37	167.02	47.55	200.2	219.68	139.21
	6−8	0	86.42	13.58	0.2	0.1	0.33	1.06	0.78	1.07	0.23	8.49	0.89	0.03	28.2	0.09	78.69	787.4	234.3	36.52	360.01	49.72	212.21	225.52	137.44
	8−10	0	86.87	13.13	0.16	0.09	0.3	1.05	0.72	0	0.16	8.4	0.85	0.03	28.16	0.08	72.13	172.18	221.42	28.68	165.5	49.65	209.12	218.04	135
	10−12	0	88.39	11.61	0.21	0.1	0.37	1.01	0.77	0	0	8.83	0.91	0	28.47	0.08	72.4	171	222.73	29.9	165.02	50.28	211.51	220.09	141.26
JM71	12−14	0	87.11	12.89	0.17	0.11	0.3	1.02	0.72	0.88	0.1	8.39	0.85	0.03	28.76	0.08	72.07	170.65	220.56	29.98	162.98	49.52	207.34	218.92	134.76
	14−16	0	87.58	12.42	0.17	0.09	0.35	1.06	0.78	1.79	0.26	8.86	0.9	0.04	29.36	0.08	72.3	176.55	227.82	31.64	163.7	49.51	213.07	223.93	141.21
	16−18	0	86.99	13.01	0.18	0.11	0.34	0.97	0.72	0.89	0.18	8.18	0.83	0	27.18	0.08	69.15	163.77	218.5	29.37	154.74	47.29	204.88	210.3	129.71
	18−20	0	84.67	15.34	0.16	0.1	0.35	1	0.74	1.02	0.41	8.42	0.85	0.03	31.38	0.08	73.23	154.12	229.16	32.82	154.29	49.6	215.18	224.44	136.01
Min		0	67.08	3.08	0.12	0.05	0.22	0.67	0.38	0	0	4.61	0.34	0	16.22	0.08	51.27	131.03	193.92	17.94	115.74	32.71	164.45	180.85	129
Max		21.26	90.41	24.45	0.34	0.12	0.59	1.44	1.25	1.79	0.65	9.69	0.97	0.04	31.38	0.11	86.44	787.4	470.55	44.95	525.11	50.28	256.95	232.83	185.67
Median		2.69	83.74	13.56	0.18	0.09	0.39	1.01	0.74	0.95	0.25	8.2	0.81	0.02	26.95	0.09	74.6	174.87	263.72	32.59	190.48	43.26	209.38	209.74	143.05
SD		6.32	5.06	4.2	0.05	0.02	0.08	0.14	0.15	0.51	0.16	1.17	0.16	0.02	3.63	0.01	7.71	120.58	51.74	7.13	80.35	6.28	18.92	15.83	13.62

	Mean square	F value	Pr(>F)
Sand	14	0.348	0.56
Silt	80	3.419	0.076
Clay	162	13.38	0.001**
Organic C	0.01	7.578	0.011*
Organic N	0.00	7.813	0.01**
Na_2O	0.02	3.275	0.082·
K_2O	0.01	0.332	0.57
CaO	0.04	1.707	0.203
MgO	0.01	0.027	0.872
Al_2O_3	0.03	1.155	0.292

Fe_2O_3	1.83	1.351	0.256
MnO	0.01	0.207	0.653
P_2O_5	0.00	2.757	0.109
As	55.9	4.843	0.037*
Cd	0.00	1.305	0.264
Co	283	5.577	0.026*
Cr	16205	1.12	0.3
Cu	3160	1.189	0.286
Mo	804	36.69	2.12e–06***
Ni	8996	1.415	0.245
Pb	135	3.713	0.065·
Sr	1.1	0.003	0.956
V	97.8	0.381	0.542
Zn	113.8	0.605	0.444
