# Supplementary material for: Microbial co-occurrence patterns and community assembly in seamount sediment cores: disentangling the effects of assembly processes on β-diversity
Source: Appl Environ Microbiol. 2026 Jun 18;92(7):e00732-26. doi: 10.1128/aem.00732-26 (PMC13390388; doi:10.1128/aem.00732-26)
Supplement: Table S4 — The significance test of the db-RDA results (the upper table) and the R2 and adjusted R2 of different groups of environmental variables for community compositional differences (the lower table). [file aem.00732-26-s0007.pdf]

Table S4 The significance test of the db-RDA results (the upper table) and the R^2 and adjusted R^2 of different groups of environmental variables for community compositional differences (the lower table).

	Df	Variance	F	Pr(>F)
Depth	1	0.031	3.883	0.001***
Clay	1	0.020	2.463	0.003**
Organic N	1	0.016	1.845	0.022*
Organic C	1	0.012	1.600	0.042*
Al_2O_3	1	0.017	2.183	0.002**
Na_2O	1	0.009	1.131	0.284
Cd	1	0.016	2.063	0.006**
Mo	1	0.010	1.254	0.190
Pb	1	0.017	2.182	0.004**
Zn	1	0.012	1.540	0.074

Significant codes: '***' for 0.001, '**' for 0.01, and '*' for 0.05.

	Df	R^2	Adjusted R^2
[a+d+f+g] = X1	1	0.105	0.070
[b+d+e+g] = X2	1	0.072	0.037
[c+e+f+g] = X3	8	0.467	0.243
[a+b+d+e+f+g] = X1+X2	2	0.171	0.105
[a+c+d+e+f+g] = X1+X3	9	0.511	0.266
[b+c+d+e+f+g] = X2+X3	9	0.497	0.246
[a+b+c+d+e+f+g] = All	10	0.541	0.271

X1: Depth;

X2: Clay;

X3: Organic N + Organic C + Al_2O_3 + Na_2O + Cd + Mo + Pb + Zn
