# Supplementary material for: Microbial co-occurrence patterns and community assembly in seamount sediment cores: disentangling the effects of assembly processes on β-diversity
Source: Appl Environ Microbiol. 2026 Jun 18;92(7):e00732-26. doi: 10.1128/aem.00732-26 (PMC13390388; doi:10.1128/aem.00732-26)
Supplement: Table S5 — Percentages of positive and negative connections among different phyla. [file aem.00732-26-s0008.pdf]

Table S5 Percentages of positive and negative connections among different phyla.

Phyla	Positive connection (%)	Negative connection (%)
Chloroflexi	19.12	22.07
γ-Proteobacteria	12.97	10.83
α-Proteobacteria	15.29	16.96
Planctomycetota	12.85	13.28
Patescibacteria	8.35	7.22
Nanoarchaeota	8.42	9.20
Bacteroidota	7.19	6.06
Acidobacteriota	6.13	6.68
Gemmatimonadota	6.63	3.27
Crenarchaeota	3.05	4.43
