# Supplementary material for: Microbial co-occurrence patterns and community assembly in seamount sediment cores: disentangling the effects of assembly processes on β-diversity
Source: Appl Environ Microbiol. 2026 Jun 18;92(7):e00732-26. doi: 10.1128/aem.00732-26 (PMC13390388; doi:10.1128/aem.00732-26)
Supplement: Table S6 — Comparing the proportions of the main phyla (%) in the six large modules with those in the entire network. [file aem.00732-26-s0009.pdf]

Table S6 Comparing the proportions of the main phyla (%) in the six large modules with those in the entire network.

Phyla	Module 1	Module 2	Module 3	Module 4	Module 5	Module 6	Entire network
Chloroflexi	12.1	13.3	15.8	2.3	11.8	6.7	11.8
γ-Proteobacteria	10.4	17.8	5.5	14.0	11.8	26.7	11.5
α-Proteobacteria	13.7	9.4	15.1	7.0	0.0	0.0	11.4
Planctomycetota	12.1	13.3	10.3	2.3	5.9	13.3	10.8
Patescibacteria	9.3	5.6	5.5	7.0	0.0	0.0	6.9
Nanoarchaeota	4.9	6.1	6.2	9.3	17.6	13.3	6.7
Bacteroidota	2.2	7.2	6.2	7.0	5.9	6.7	5.6
Acidobacteriata	3.8	6.1	4.8	9.3	5.9	0.0	5.3
Gemmatimonadota	3.8	0.6	4.1	9.3	0.0	13.3	3.1
Crenarchaeota	4.4	4.4	1.4	2.3	0.0	0.0	3.1
