# Supplementary material for: Microbial co-occurrence patterns and community assembly in seamount sediment cores: disentangling the effects of assembly processes on β-diversity
Source: Appl Environ Microbiol. 2026 Jun 18;92(7):e00732-26. doi: 10.1128/aem.00732-26 (PMC13390388; doi:10.1128/aem.00732-26)
Supplement: Table S7 — Numbers of module hubs, connectors, and peripherals in the six large modules. [file aem.00732-26-s0010.pdf]

Table S7 Numbers of module hubs, connectors, and peripherals in the six large modules.

Categories	Module 1	Module 2	Module 3	Module 4	Module 5	Module 6
Module hubs	5	6	0	1	1	0
Connectors	10	7	1	0	0	1
Peripherals	167	167	145	42	16	14
